# *Acanthopanax senticosus* Promotes Survival of Tilapia Infected With *Streptococcus iniae* by Regulating the PI3K/AKT and Fatty Acid Metabolism Signaling Pathway

**DOI:** 10.3389/fphys.2021.699247

**Published:** 2021-07-09

**Authors:** Hong Xia Li, Jun Qiang, Chang You Song, Pao Xu

**Affiliations:** ^1^Wuxi Fisheries College, Nanjing Agricultural University, Wuxi, China; ^2^Key Laboratory of Freshwater Fisheries and Germplasm Resources Utilization, Ministry of Agriculture and Rural Affairs, Freshwater Fisheries Research Center, Chinese Academy of Fishery Sciences, Wuxi, China

**Keywords:** *Acanthopanax senticosus*, genetic improvement of farmed tilapia, *Streptococcus iniae*, metabolism, immune response

## Abstract

*Streptococcus* has greatly restricted the development of healthy tilapia aquaculture. As a green and efficient feed addition, *Acanthopanax senticosus* (APS) has been increasingly used in culture, but it is unclear whether it represents a disease-resistant feed. Genetically improved farmed tilapia (GIFT, *Oreochromis niloticus*) was fed with a feed supplemented with 0 (control), 0.5, 1, 2, 4, and 8‰ APS for 56 days, after which fish were injected with 5.9 × 10^6^ CFU/ml *Streptococcus iniae* into the abdominal cavity. At 96 h after infection, the cumulative survival of GIFT in control and 0.5‰ APS treatments was significantly lower than in other treatments; at APS supplementation rates of 1 and 2‰, serum glucose, triglycerides, and cholesterol contents were all significantly lower than in control treatment fish. Hepatic glycogen and triglyceride contents of 1‰ APS treatment fish were significantly higher than those in fish in control treatment. Transcription levels of peroxisome proliferator activated receptor α (PPAR), fatty acid synthase (FAS), and lipoprotein Lipase (LPL) genes were upregulated, and their expression levels in fish in 1, 2, and 4‰ treatments were significantly higher than those in fish in control treatment at 96 h after *S. iniae* infection. After 96 h of infection, the red blood cells, hemoglobin, hematocrit, and white blood cells of fish in 1‰ APS treatment were significantly lower than those of fish in 4 and 8‰ treatments; hepatic catalase activity was activated at 48 h, superoxide dismutase activity was also significantly upregulated at 96 h, and the malondialdehyde content significantly decreased. It is noted that 0.5–2‰ APS treatments significantly activated the expression of PI3K and AKT in the liver, while inhibiting the expression of Caspase-9. Therefore, feed with 1‰ APS can promote hepatic glycogen and lipid metabolism in GIFT after infection with *S. iniae*, which is beneficial to alleviating oxidative stress damage and cell apoptosis in liver tissue.

## Introduction

The risk of disease has increased with the rapid development of tilapia aquaculture, with *Streptococcus* being one of the biggest threats to their healthy farming. The main pathogens are *Streptococcus iniae* and *Streptococcus agalactiae* (Verner-Jeffreys et al., 2018; Abdel-Latif et al., 2020). Disease from *S. iniae* spreads fast and causes considerable losses to aquaculture industries in warm water areas due to increased mortalities; it infects at least 27 marine and freshwater fish species at mortality rates as high as 30–50% (Abdel-Latif et al., 2020). The frequency of streptococcal disease is related to the stress response of fish caused by the mode of aquaculture and physical and chemical attributes of the water quality (Qiang et al., 2016; Abdel-Latif et al., 2020).

Siberian ginseng *Acanthopanax senticosus* (APS) is rich in nutrients such as trace elements and amino acids, as well as active ingredients such as saponins, polysaccharides, and flavonoids (Yi et al., 2001). We earlier reported that feed supplemented with APS significantly reduced hepatic fat deposition in genetically improved farmed tilapia (GIFT, *Oreochromis niloticus*) and hybrid yellow catfish (*Tachysurus fulvidraco*♀ × *Pseudobagrus vachellii*♂) and improved their growth performance and non-specific immunity (Li H. et al., 2021; Li M.X. et al., 2021). As a traditional Chinese herbal medicine, APS is safe, environmentally friendly, and non-drug resistant. Many studies have confirmed that APS can actively enhance immunity, antioxidant capacity, and resistance to pathogen invasion (Gaffney et al., 2001; Guo et al., 2006; Chen et al., 2011), improve intestinal microflora (Kong et al., 2017), and treat inflammation in animals (Yamazaki et al., 2007). The signaling pathway involved in PI3K/AKT plays an important role in bacteria induced in immune response and cell cycle functions. Largemouth bass (*Micropterus salmoides*) can activate the PI3K/AKT pathway to regulate the proliferation of immune cells and thus reduce inflammation in response to *Aeromonas hydrophila* infection (Zhou et al., 2021). Also, feed supplemented with methionine, oxidized fish oil, or high sugar can regulate the antioxidant and proinflammatory response of fish through the PI3K/AKT pathway (Pan et al., 2017; Song et al., 2018; Ke et al., 2020).

Genetically improved farmed tilapia is currently one of the mainly cultured freshwater fish species in China because of their fast growth and high yield. As consumers are increasingly concerned about food safety, the study on the development of green and healthy antibiotic substitute products has increased (Cabello, 2006). No safe and efficient drugs for treating tilapia *Streptococcus* are known. Drug susceptibility tests of *S. iniae* on tilapia have demonstrated that Chinese herbal medicines such as honeysuckle *Lonicerae Japonicae Flos*, Chinese Goldthread *Coptidis Rhizoma*, comfrey *Symphytum officinale*, and gallnut *Galla Chinensis* have strong antibacterial and bactericidal effects (Hua, 2002; Ying-Rui et al., 2013; Gabriel et al., 2015). Therefore, to determine if APS improves the response and regulation mechanism of GIFT infected with *S. iniae*, we compared their serum biochemistry, liver antioxidant, lipid metabolism, and PI3K/AKT pathways after feeding GIFT with different APS levels.

## Materials and Methods

### Experimental Fish

Fish were obtained from the Freshwater Fisheries Research Center, Chinese Academy of Fishery Sciences and acclimated in an indoor water-cycling system under natural light, with dissolved oxygen (DO) concentration held at 5.82 mg/l ± 0.45, temperature at 28°C ± 0.7, and pH at 7.76 ± 0.02, for 2 weeks. Each day at 8:00 and 16:30, fish were fed a floating compound feed (30% protein and 6% fat) at about 8% of their body mass.

### Experiment Management and Design

*Acanthopanax senticosus* ultrafine powder was added to the basic feed at amounts of 0 (control), 0.5, 1.0, 2.0, 4.0, and 8.0‰ (for formula and production of specific experimental diets, see Li H. et al., 2021).

After acclimation, 720 GIFT with no obvious signs of disease or injury were selected and weighed (using an electronic balance), with an inclusion criterion for this experiment being that they were within 0.02 g of an average weight of 6.50 g. Of these, 40 fish were randomly placed into each of 18 circulating water tanks of 800 l; for each treatment, there were 3 replicates [40 fish for each of three replicates per treatment (120 fish per treatment) × 6 treatments = 720 fish].

Any fish that died in the first week of experimentation were replaced with comparably sized fish. Fish in all treatments were fed close to satiation two times daily, at 08:00 and 17:00. Water quality was monitored daily; water temperature ranged 26–28°C, DO exceeded 5.0 mg/l, and ammonia nitrogen and nitrite were lower than 0.02 and 0.03 mg/l, respectively. The experiment was carried out for 8 weeks.

Fish were initially fasted for 24 h at the end of experimentation; 10 fish were randomly selected earlier from each tank, transferred to a 200 l tank, and maintained in similar conditions to appraise postinfection mortality; remaining fish in each tank were reserved for the postinfection analysis of serum and hepatic biochemistry and molecular biology. Fish in each treatment were injected intraperitoneally with *S. iniae* of 5.9 × 10^6^ CFU/ml (Gabriel et al., 2015). Survival and the parameters of each treatment at 0 (before infection), 24, 48, and 96 h (hours postinfection, hpi) were identified (for culture and concentration determination of *S. iniae*, see Qiang et al., 2017).

### Treatment and Sampling

Fish were deeply anesthetized with MS-222, after which 2.0 ml syringes were used to draw blood through the tail vein. Blood samples were placed in 1.5 ml EP tubes, from which 0.2 μl samples were drawn for analysis. Residual blood was placed in a refrigerator at 4°C for 2 h, and then the upper serum was separated by centrifugation at 5,000 *g*. The liver tissue was dissected into three equal parts on a disinfected platform: two parts were placed into two 1.5 ml cryotubes, quickly frozen with liquid nitrogen, and stored in a refrigerator at −80°C and the third part was placed in 4% paraformaldehyde for histological analysis.

### Biochemical Analyses

We measured red blood cells (RBC), white blood cells (WBC), hemoglobin (Hb), and hematocrit (Hct) using an automatic blood analyzer (TG, Mindray, Shenzhen, China). Serum glucose (Glu), alanine aminotransferase (AST), aspartate aminotransferase (ALT), triglycerides (TC), and cholesterol (TG) were measured using a fully automatic biochemical analyzer (BS-800, Mindray, Shenzhen, China). All kits were purchased from Mindray, Shenzhen, China.

One liver sample was homogenized at a ratio of 9 ml of physiological saline per gram of tissue (100 g/l homogenates) in an ice-water bath and centrifuged at 2,000 *g* at 4°C for 10 min. The supernatant was then stored in a refrigerator at 4°C. The enzyme-linked immunosorbent assays were used to measure superoxide dismutase (SOD), catalase (CAT), malondialdehyde (MDA), complement C3, lysozyme, glycogen, TG, TC, and protein content, with kits purchased from Shanghai Haoben Biotechnology Co., Ltd. (Shanghai, China). The results were read on a full-band microplate reader (BioTek Eon, Winooski, United States). By measuring the absorbance of each group of samples at different wavelengths, the corresponding sample concentrations could be calculated according to the standard curve of each detection index.

### Detecting Cell Apoptosis by the Terminal Deoxynucleotidyl Transferase dUTP Nick End Labeling Method

After fixation for 24 h, liver tissue samples were dehydrated and transparent, embedded in paraffin, and sectioned. After deparaffinization with xylene, terminal deoxynucleotidyl transferase dUTP nick end labeling (TUNEL) staining (Qiang et al., 2021) was performed, and the samples were observed and photographed using a NIKON E100 imaging system (NIKON DS-U3, Japan).

### Analysis of Gene Expression

Total RNA from liver tissue was extracted using TRIzol reagent (Invitrogen, Carlsbad, United States). According to the instructions of PrimeScript^TM^ RT Master Mix reverse-transcription kit (Takara, Dalian, China), mRNA was reverse-transcribed to cDNA. The operating steps of SYBR Premix Ex Taq kit were followed to detect gene expression separately. β-Actin was used as an internal reference gene. The Applied Biosystems QuantStudio 5 Real-Time PCR System (Applied Biosystems, Foster City, CA, United States) was used to detect the mRNA expression level of related genes. Primer synthesis was performed by Suzhou Jinweizhi Biotechnology Co., Ltd. (Suzhou, China) (Table 1). The 20 μl reaction system comprised 0.6 μl of each forward and reverse primer (10 mmol/μl), 1 μl of cDNA, 10 μl of 2 × SYBR Premix Ex Taq II, and 7.8 μl of RNase-free water. Reaction conditions included 95°C for 5 min, 40 cycles (95°C for 15 s and 60°C for 60 s), and finally a dissolution reaction, including 95°C for 15 s, 60°C for 15 s, and 95°C for 15 s. Each reaction consists of three sets of repetitions. All test samples contained a negative control without templates to rule out false-positive results.

**TABLE 1:** The primer sequence of the gene in this experiment.

**Gene abbreviation**	**Sequence**
FAS	F: 5′-CAGCTGCAGACCCAGAATCA-3′
	R: 5′-AATGCCAGTGAACTGCTGGA-3′
LPL	F: 5′-TTGCCGGAGACCTTACCAAC-3′
	R: 5′-AGGACATCCACAAACTGGGC-3′
PPAR-α	F: 5′-TAGCAAGCCGCCTTTCATCA-3′
	R: 5′-AGTCTCCACTGAGGTGCTCT-3′
PI3K	F: 5′- ATCCTCAAAGTCTGCGGCTG -3′
	R: 5′-CCAGCATGATGCAACTACGG-3′
AKT	F: 5′- CGTCCTCCCAGCAATCGTCT-3′
	R: 5′-CCACACAGGTATGTTGTCTCGG-3′
Caspase-9	F: 5′-CTATTCCTACGCCTGACGGG-3′
	R: 5′-GCCCTTGCGATTTTTCAGCA-3′
β-actin	F: 5′-CCACACAGTGCCCATCTACGA-3′
	R: 5′-CCACGCTCTGTCAGGATCTTCA-3′

### Data Analysis

Data were expressed as means ± standard errors, with SPSS ver. 22.0 (IBM Corp., Armonk, NY, United States) software used for statistical analyses. The Shapiro–Wilk and Levene’s tests were used to analyze the normality and homogeneity of data, and the one-way ANOVA was used to analyze the statistical significance. When the data obtained were statistically significant, a Duncan’s test was used for multiple comparisons. *P* < 0.05 is considered statistically significant.

## Results

### Effect of APS Feed Supplement on Survival of GIFT Infected With *S. iniae*

**FIGURE 1:**
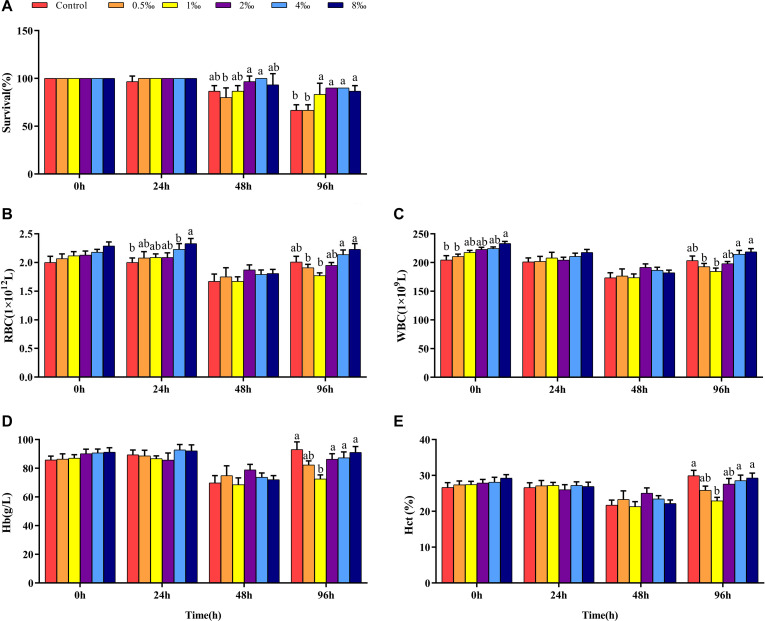
Survival and blood parameters of genetically improved farmed tilapia (GIFT) infected with *Streptococcus iniae* after being fed different *Acanthopanax senticosus* (APS) levels. **(A–E)** Represent the control, 0.5, 1, 2, 4, and 8‰ APS supplementation treatments, respectively. Different lowercase letters show significant differences among different treatments at each sampling point (Duncan’s multiple range test; *P* < 0.05).

Genetically improved farmed tilapia began to die after 24 h of being infected with *S. iniae*, but there was no significant difference in mortality among treatments (*P* > 0.05) (Figure 1A). At 48 hpi, GIFT death occurred in all treatments except for 4‰ treatment. The cumulative survival of GIFT in 0.5‰ treatment was significantly lower than that in fish in 2 and 4‰ treatments (*P* < 0.05). At 96 hpi, the cumulative survival of GIFT in control and 0.5‰ treatments was significantly lower than for other APS treatments.

### Effect of APS Feed Supplement on Blood Parameters of GIFT Infected With *S. iniae*

Before infection, the number of RBC in fish in each treatment did not differ significantly (*P* > 0.05). As infection time increased, the number of RBC in fish in 4 and 8‰ treatments was significantly higher than that in fish in control treatment (*P* < 0.05) (Figure 1B). At 48 hpi, there was no significant difference between control and treatment fish, but at 96 hpi, the number of RBC in fish in 0.5 and 1‰ treatments was significantly lower than those in 4 and 8‰ treatments. Before infection, the number of WBC in fish in 8‰ treatment was significantly higher than that in fish in control and 0.5‰ treatments. At 96 hpi, the number of WBC in fish in 0.5 and 1‰ treatments was significantly lower than that in 4 and 8‰ treatments (Figure 1C). There was no significant difference in Hb and Hct in treatment fish before or at 24 and 48 hpi. However, at 96 hpi, Hb and Hct in fish in 1‰ treatment were significantly lower than those in fish in control, 4 and 8‰ treatments (*P* < 0.05) (Figures 1D,E).

### Effect of APS Feed Supplement on Serum Biochemical Parameters of GIFT Infected With *S. iniae*

**FIGURE 2:**
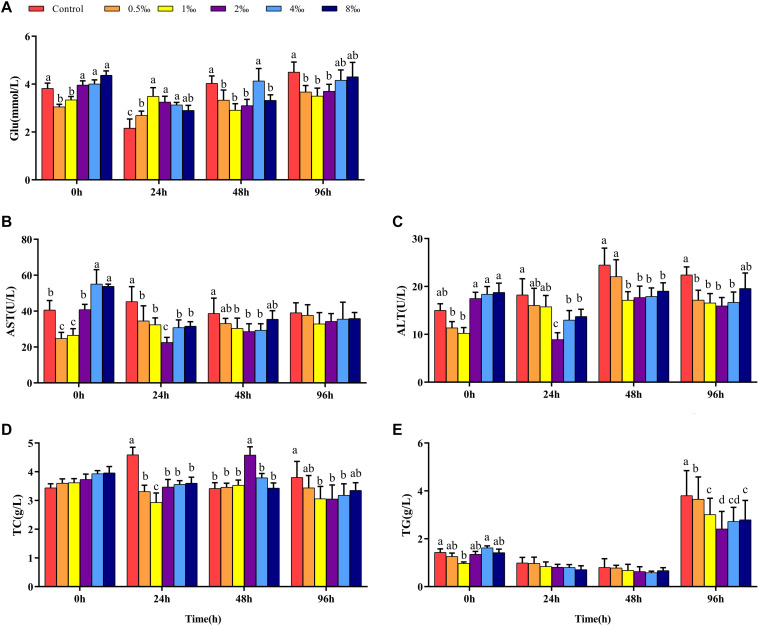
Serum parameters in GIFT infected with *S. iniae* after being fed different APS levels. **(A–E)** Represent the control, 0.5, 1, 2, 4, and 8‰ APS supplementation treatments, respectively. Different lowercase letters show significant differences among different treatments at each sampling point (Duncan’s multiple range test; *P* < 0.05).

Before infection, fish in 0.5 and 1‰ treatments had significantly lower Glu contents than control, 2, 4, and 8‰ APS treatment fish (Figure 2A). At 24 hpi, the Glu content in control and 0.5‰ treatment fish was significantly lower than in 1, 2, and 4‰ treatment fish. However, at 48 and 96 hpi, the Glu content in fish in 1 and 2‰ treatments was significantly lower than that in control treatment fish (*P* < 0.05). Before infection, the AST and ALT activities in fish in 0.5 and 1‰ treatments were significantly lower than those in other treatments. At 48 hpi, the AST and ALT activities in fish in 1, 2, and 4‰ treatments were significantly lower than those in fish in control treatment (Figures 2B,C). There was no significant difference in the serum TC content of fish in any treatment before infection. As the infection time increased, the serum TC content of fish in 2‰ treatment was significantly higher than that in fish in other treatments at 48 hpi (*P* < 0.05) (Figure 2D). At 96 hpi, the serum TC and TG levels (Figure 2E) in fish in control treatment were significantly higher than those in fish in 1, 2, and 4‰ treatments.

### Effect of APS Feed Supplement on Hepatic Biochemical Parameters of GIFT Infected With *S. iniae*

**FIGURE 3:**
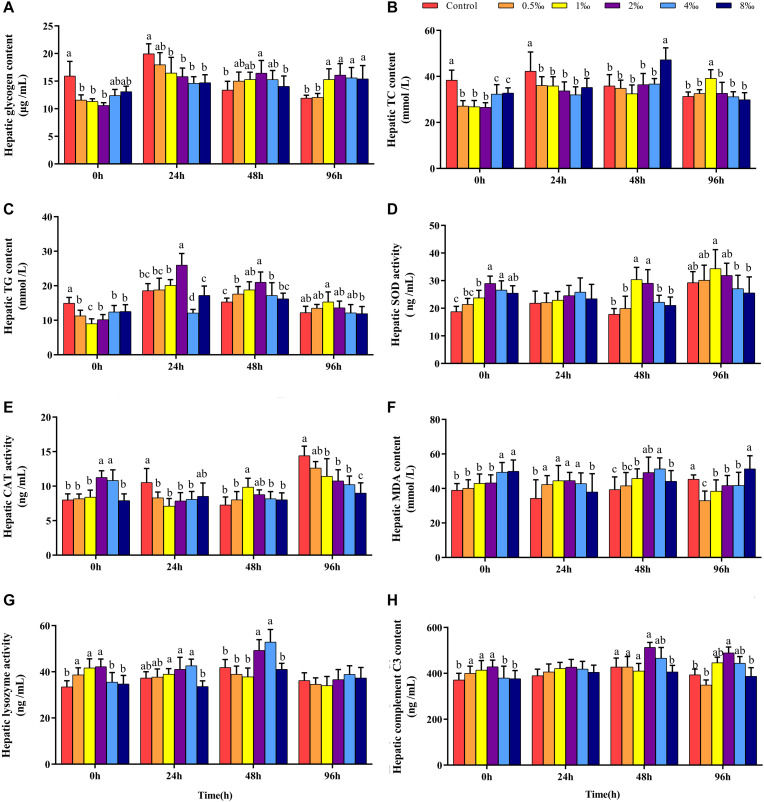
Hepatic biochemical parameters of GIFT infected with *S. iniae* after being fed different APS levels. **(A–H)** Represent the control, 0.5, 1, 2, 4, and 8‰ APS supplementation treatments, respectively. Different lowercase letters show significant differences among different treatments at each sampling point (Duncan’s multiple range test; *P* < 0.05).

Figures 3A–C show that before infection, hepatic glycogen, TC, and TG contents of fish in 0.5, 1, and 2‰ treatments were significantly lower than those in control treatment fish (*P* < 0.05). With increased infection time, at 48 hpi, the hepatic glycogen content of fish in 2‰ treatment was significantly higher than that in fish in control and 8‰ treatments. At 96 hpi, the hepatic glycogen content in fish in 1–8‰ treatments was significantly higher than that in fish in control and 0.5‰ treatments. At 96 hpi, the hepatic TC content of fish in 1‰ treatment was significantly higher than that in fish in all other treatments, and the TG content was significantly higher than that in fish in 8‰ treatment.

Before infection, hepatic SOD and CAT activities in fish in 2 and 4‰ treatments were significantly higher than those in fish in control, 0.5 and 1‰ treatments (Figures 3D,E; *P* < 0.05). At 48 hpi, hepatic SOD activities in fish in 1 and 2‰ treatments were significantly higher than those in fish in other treatments; at 96 hpi, hepatic SOD activity in fish in 1‰ treatment was significantly higher than that in fish in 4 and 8‰ treatments. At 48 hpi, hepatic CAT activity in fish in 1‰ treatment was significantly higher than that in fish in other treatments; however, at 96 hpi, with increased APS levels, hepatic CAT activity declined significantly. Before infection, the hepatic MDA content in fish in 4 and 8‰ treatments was significantly higher than that in fish in other treatments; at 96 hpi, the MDA content in fish in 0.5–4‰ treatments was significantly lower than that in fish in control and 8‰ treatments. Before infection, hepatic lysozyme and complement C3 (Figure 3F) contents in fish in 0.5–2‰ treatments were significantly higher than those in fish in control, 4 and 8‰ treatments (Figures 3G,H); with extended infection time, the lysozyme content in fish in 2 and 4‰ treatments were significantly higher than in fish in other treatments at 48 hpi. The complement C3 content of fish in 2‰ treatment was significantly higher than that in fish in control, 0.5 and 8‰ treatments at 48 and 96 hpi (*P* < 0.05).

### Effect of APS Feed Supplement on Fatty Acid Metabolism Signaling Pathways in GIFT Infected With *S. iniae*

**FIGURE 4:**
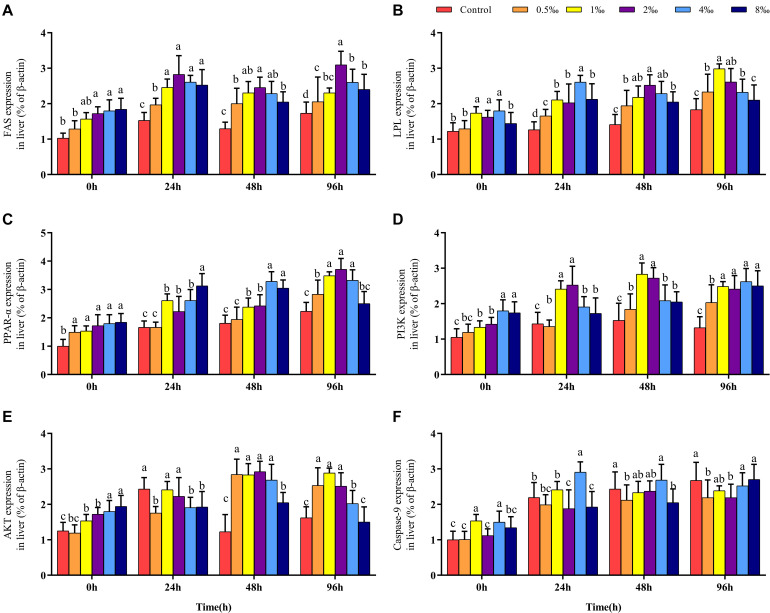
Fatty acid metabolism and PI3K/AKT pathway of GIFT infected with *S. iniae* after being fed different APS levels. **(A–F)** Represent the control, 0.5, 1, 2, 4, and 8‰ APS supplementation treatments, respectively. Different lowercase letters show significant differences among different treatments at each sampling point (Duncan’s multiple range test; *P* < 0.05).

Before infection, the expression of the FAS gene in the liver of fish in 2–8‰ treatments was significantly higher than that in fish in control, 0.5 and 1‰ treatments. After *S. iniae* infection, the expression level of FAS in the liver of fish in each treatment trended upward; the expression level of FAS in fish in 2‰ treatment was significantly higher than that in fish in control and 0.5‰ treatments at 24, 48, and 96 hpi (*P* < 0.05) (Figure 4A). Before infection, the expression levels of LPL and PPAR-α in fish in 0.5–4‰ treatments were significantly higher than those in fish in control, 0.5 and 8‰ treatments (Figures 4B,C). At 24, 48, and 96 hpi, the expression level of LPL in fish in 0.5–4‰ treatments was significantly higher than that in fish in control treatment; the expression level of PPAR-α in fish in 0.5–4‰ treatments was significantly higher than that in fish in control and 0.5‰ treatments (*P* < 0.05).

### Effect of APS Feed Supplement on PI3K/AKT Signaling Pathway in GIFT Infected With *S. iniae*

Before infection, as the APS content increased, the hepatic PI3K and AKT gene expression levels also gradually increased; gene expression levels in fish in 4 and 8‰ treatments were significantly higher than those in fish in control and 0.5‰ treatments. At 48 and 96 hpi, expression levels of PI3K in the liver of fish in 1 and 2‰ treatments were significantly higher than those in fish in control and 0.5‰ treatments (*P* < 0.05) (Figures 4D,E); the expression levels of AKT in fish in 0.5–4‰ treatments were significantly higher than those in fish in control and 8‰ treatments. The expression level of the Caspase-9 gene in the liver of fish in 4‰ treatment was significantly higher than that in fish in 0.5 and 8‰ treatments before infection and at 24 and 48 hpi. However, at 96 hpi, the expression levels of Caspase-9 in fish in control and 8‰ treatments were significantly higher than those in fish in 0.5 and 2‰ treatments (*P* < 0.05) (Figure 4F).

### Effect of APS Feed Supplement on Hepatocyte Apoptosis in GIFT Infected With *S. iniae*

**FIGURE 5:**
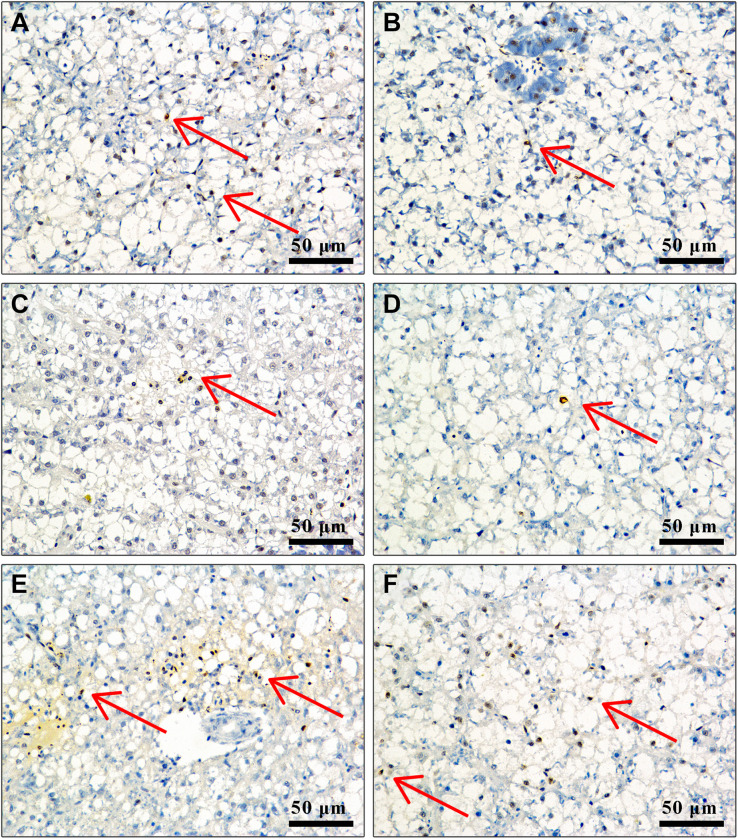
Liver cell apoptosis of GIFT infected with *S. iniae* after being fed different APS levels. **(A–F)** Represent the control, 0.5, 1, 2, 4, and 8‰ APS supplementation treatments, respectively. The red arrow indicates apoptotic cells.

In TUNEL-stained cells, hematoxylin stains the nuclei of normal hepatocytes in blue color and those of positive apoptotic cells in irregular brown color. Figure 5 shows that the number of positive apoptotic cells in fish in control, 0.5, 4, and 8‰ treatments is significantly higher than in fish in 1 and 2‰ treatments at 96 hpi.

## Discussion

Traditional Chinese herbal medicines or their components have recently been widely used as immunostimulants in aquaculture (Dawood et al., 2018). As a feed additive for GIFT, APS plays an important role in boosting the immune system, increasing antioxidant capacity, and improving pathogen resistance (Gaffney et al., 2001; Guo et al., 2006). We reported a reduced mortality of GIFT at 96 h after *S. iniae* infection when their food was supplemented with APS above 1‰, but how this occurs remain unclear. We further investigated the effects of APS on GIFT lipid metabolism, immune response, and anti-oxidation after infection with *S. iniae*.

Blood is an important transport system within the fish body, and it is involved in regulating glucose and lipid metabolism. The lipid content of blood can indicate the levels of fat metabolism in fish, with decreased serum TC and TG contents indicating increased hepatic lipid metabolism (Qiang et al., 2018). Serum TG and Glu are preferentially used to provide energy under acute stress, such as the energy associated with bacterial infection and hypoxia (Qiang et al., 2012, 2015; Sheng et al., 2019). We reported that 96 h following infection with *S. iniae*, serum Glu, TC, and TG levels in fish in 1 and 2‰ treatments were significantly lower than those in fish in control treatment. Additionally, Glu and TC contents in liver tissues of fish in 1‰ treatment were significantly higher than those in fish in control treatment; the TG content was also slightly higher. Fish infected with *S. iniae* in 1‰ treatment had elevated hepatic glycogen and lipid metabolisms, providing sufficient energy to resist bacteria. However, an excessive addition of APS may weaken glucose and lipid metabolism in fish and increase serum Glu, TC, and TG.

As a family of nuclear hormone receptors, PPARs primarily play a regulatory role in lipid metabolism, fat formation, and the maintenance of metabolic stability (Michalik et al., 2006; Dunning et al., 2014). FAS is a key catalyst in fatty acid synthesis and participates in the production of endogenous ligands for PPAR-α (Merkel et al., 2002; Chakravarthy et al., 2009). LPL is a key catalyst in the decomposition of TG, which is regulated by PPAR-α (Merkel et al., 2002). We reported that after *S. iniae* infection, the expression levels of PPAR-α, FAS, and LPL in each treatment were all upregulated, and the levels of these genes in fish in 1, 2, and 4‰ treatments were significantly higher than those in fish in control treatment. Luo et al. (2019) also reported that PPAR-α activation could increase the mitochondria-mediated energy supply and enhance the ability of Nile tilapia to resist the infection by *Aeromonas hydrophila*. Therefore, adding APS may assist with the activation of hepatic lipid metabolism pathways, promote lipid catabolism, and provide more energy to ward off bacterial infection.

After being infected by bacteria, fish not only metabolize lipids but also respond to pathogenic invasion through their immune system. Some blood parameters (i.e., RBC, Hb, Hct, and WBC) can be used to assess the physiological and pathological conditions of fish (Harikrishnan et al., 2011). In blood, RBC plays an important role in transporting oxygen and carbon dioxide, while Hb is a special protein that binds to oxygen in RBC, with a concentration closely related to the RBC content (Whitehead et al., 2019). Hct refers to the proportion of RBC in blood, mainly affecting blood viscosity, and also indicates oxygen transport capacity (Bao et al., 2018). Changes in Hb and Hct may be related to the nutritional status of fish. It is noted that 1‰ treatment may promote efficient utilization of nutrients, reducing the metabolic burden. Fish in 1‰ treatment might also require less oxygen to transport nutrients, reducing RBC, Hb, and Hct levels.

White blood cell is a type of blood cell involved in non-specific immune defense. When fish are infected by pathogens or under stress, the number of WBC in the blood changes (Qiang et al., 2013; Ma et al., 2015). We reported that at 96 hpi, the WBC counts in fish in 0.5 and 1‰ treatments were significantly lower than those in fish in 4 and 8‰ treatments. We speculated that when compared with 0.5 and 1‰ treatments, other GIFT treatments need higher non-specific immunity to promote protection when combating infection. The ALT and AST activities in serum are sensitive indicators of liver damage or injury from various stresses (Qiang et al., 2015). Serum ALT and AST activities in fish in 1‰ treatment were significantly lower than those in fish in 0‰ treatment within 48 hpi, indicating that an APS supplement of 1‰ can play a role in liver protection. Complement C3 is a glycoprotein with similar enzymatic activity; it is synthesized by hepatocytes and macrophages and plays an important role in resisting pathogenic invasion and inflammation. The complement system is an indispensable part of the non-specific immune system of the body, and its follow-up response is inseparable from the activation of complement C3 (Smith et al., 2001). In this study, the level of complement C3 in the liver of fish in 1‰ treatment increased, indicating that APS may stimulate complement C3 to participate in the regulation of non-specific immunity of fish exposed to *S. iniae* infection.

In normal physiological metabolism, the antioxidant system can promptly eliminate oxygen free radicals produced in the body. However, when a body is under stress, if many oxygen free radicals are not cleared in time, oxidative damage to cells will occur (Franco et al., 2009). SOD and CAT are key enzymes in the antioxidant system. In the process of removing reactive oxygen species, O^2–^ is disproportionated by SOD into H_2_O_2_ and H_2_O, and CAT can further reduce the product H_2_O_2_ to H_2_O. Therefore, the effects of SOD and CAT on the body can reduce the oxidative damage of O^2–^ to cells (Jiang et al., 2016). We reported the hepatic CAT in fish in 1‰ treatment was activated at 48 hpi, and the SOD activity in the liver was significantly upregulated at 96 hpi. These results suggest that 1‰ treatment may activate the oxidative defense system of GIFT to remove excess oxygen when infected.

Malondialdehyde is an important substance produced by the attack of oxygen free radicals on polyunsaturated fatty acids on biofilms. It can cause the cross-linking and polymerization of biological macromolecules, such as nucleic acids and proteins, causing cell damage. The MDA content indirectly reflects the level of active oxygen and lipid peroxidation in the body and the degree of cell damage (Luder et al., 2003). We reported that the content of MDA in fish in 0.5–4‰ treatments was significantly lower than that in fish in control treatment at 96 hpi, indicating that feed supplemented with an appropriate amount of APS may help alleviate the oxidative stress damage following *S. iniae* infection.

Apoptosis refers to the orderly and autonomous death of cells controlled by genes to maintain the stability of the internal environment. Apoptosis is closely related to immune regulation in fish. Through TUNEL-stained sections, we found that fish in 1 and 2‰ treatments had a weaker degree of apoptosis, suggesting that supplementation with APS alleviated apoptosis of liver tissue following *S. iniae* infection. To explore the regulation mechanism of APS on cell apoptosis of GIFT exposed to *S. iniae* infection, we identified the PI3K/AKT pathway-related gene expression. PI3K/AKT is an important signal transduction pathway in animals, which participates in cell apoptosis directly or indirectly by regulating apoptosis-related factors (Yang et al., 2008). AKT is a key downstream mediator of PI3K. Activated PI3K converts membrane-bound phosphatidylinositol 4,5-bisphosphate (PIP2) into phosphatidylinositol 3,4,5-triphosphate (PIP3), with PIP3 acting as a second messenger in a cell that can bind to and activate the signal protein AKT downstream of PI3K, thereby exerting an anti-apoptotic effect (Zhu et al., 2015). We reported that following *S. iniae* infection, fish in 0.5–2‰ treatments had significantly activated the expression of PI3K and AKT transcription levels in the liver and inhibited the expression of the apoptotic protein Caspase-9. This result is consistent with the findings observed by section staining, suggesting that the PI3K/AKT pathway may be involved in alleviating hepatocyte apoptosis when GIFT are infected with *S. iniae*.

## Data Availability Statement

The original contributions presented in the study are included in the article/Supplementary Material, further inquiries can be directed to the corresponding author/s.

## Ethics Statement

The study protocols and design were approved by the Ethics Committee at the Freshwater Fisheries Research Center of the Chinese Academy of Fishery Sciences (Wuxi, China). Written informed consent was obtained from the owners for the participation of their animals in this study.

## Author Contributions

PX conceived and designed the experiments. HL, JQ, and CS conducted *S. iniae* infection experiments, collected samples, extracted RNA, and conducted qRT-PCR experiments. HL conducted biochemical analyses and wrote the manuscript with contributions from all other authors. JQ and CS analyzed the data. All authors have read and approved the final version of this manuscript.

## Conflict of Interest

The authors declare that the research was conducted in the absence of any commercial or financial relationships that could be construed as a potential conflict of interest.
